# Exploring previously used thresholds for computed tomography‐defined low skeletal muscle mass in predicting functional limitations among lung cancer patients

**DOI:** 10.1111/1759-7714.15313

**Published:** 2024-04-26

**Authors:** Shiva Raj Timsina, Wiwatana Tanomkiat, Sarayut L. Geater, Natee Ina

**Affiliations:** ^1^ Department of Radiology, Faculty of Medicine Prince of Songkla University Songkla Thailand; ^2^ Unit of Respiratory and Respiratory Critical Care Medicine, Department of Medicine, Faculty of Medicine Prince of Songkla University Songkla Thailand

**Keywords:** lung cancer, physical functions, sarcopenia, skeletal muscle mass, SPPB

## Abstract

**Background:**

Various cutoffs have been used to diagnose computed tomography (CT)‐defined low skeletal muscle mass; however, the impact of this variability on predicting physical functional limitations (PFL) remains unclear. In the present study we aimed to evaluate the diagnostic test metrics for predicting PFLs using a fixed cutoff value from previous reports and sought to create a prediction score that incorporated the skeletal muscle index (SMI) and other clinical factors.

**Methods:**

In this cross‐sectional study including 237 patients with lung cancer, the SMI was assessed using CT‐determined skeletal muscle area at the third lumbar vertebra. Physical function was assessed using the short physical performance battery (SPPB) test, with PFL defined as an SPPB score ≤9. We analyzed the diagnostic metrics of the five previous cutoffs for CT‐defined low skeletal muscle mass in predicting PFL.

**Results:**

The mean age of participants was 66.0 ± 10.4 years. Out of 237 patients, 158 (66.7%) had PFLs. A significant difference was observed in SMI between individuals with and without PFLs (35.7 cm^2^/m^2^ ± 7.8 vs. 39.5 cm^2^/m^2^ ± 8.4, *p* < 0.001). Diagnostic metrics of previous cutoffs in predicting PFL showed suboptimal sensitivity (63.29%–91.77%), specificity (11.39%–50.63%), and area under the receiver operating characteristic curve (AUC) values (0.516–0.592). Age and the SMI were significant predictors of PFL; therefore, a score for predicting PFL (age – SMI + 21) was constructed, which achieved an AUC value of 0.748.

**Conclusion:**

Fixed cutoffs for CT‐defined low skeletal muscle mass may inadequately predict PFLs, potentially overlooking declining physical functions in patients with lung cancer.

## Graphical abstract

The diagnostic test metrics of five previous cutoffs for computed tomography (CT)‐defined low skeletal muscle index (SMI) in predicting physical functional limitations (PFLs) were determined. The cutoffs exhibit suboptimal sensitivity, specificity and overall accuracy. A simple prediction score combining age and SMI demonstrated enhanced predictive ability. Previous CT‐defined low SMI cutoffs may inadequately predict PFLs, potentially missing declining physical functions.

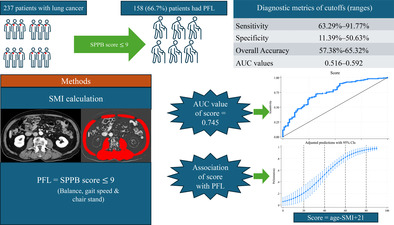

## INTRODUCTION

Lung cancer ranked second in terms of global cancer diagnoses in 2020, and contributed to 18% of cancer‐related deaths worldwide.^1^ In lung cancer, the stage of the disease is a crucial determinant of outcomes, but the presence of sarcopenia, affecting 43% of patients, is increasingly recognized for its negative effects on prognosis, treatment complications, physical function, and survival.^2^, ^3^, ^4^, ^5^, ^6^, ^7^ Low skeletal muscle mass is a diagnostic criterion for sarcopenia along with muscle strength and physical performance.^8^, ^9^ Computed tomography (CT) is the preferred method for noninvasively measuring muscle mass in patients with cancer. The cross‐sectional muscle area at the level of the third lumbar vertebrae (SMA) has been established as a reliable indicator of the estimated total body muscle mass.^8^, ^10^

Skeletal muscle mass shows an expedited decline in cancer patients as compared with the normal age‐related decline in individuals without cancer.^11^ This rapid decline has an array of negative outcomes that are increasingly recognized in lung cancer patients.^2^, ^3^, ^4^, ^5^, ^6^, ^7^ In addition to a decline in skeletal muscle mass, cancer patients also exhibit a decline in other parameters of sarcopenia, such as muscle strength and physical performance, which leads to physical functional impairment.^5^, ^11^

Physical performance can be assessed through tests such as the short physical performance battery (SPPB), which offers a reliable performance‐based evaluation of physical function and enables appropriate measurement of changes in functional limitations over time.^12^, ^13^ Moreover, the SPPB test has the potential to predict the completion of chemotherapy and postoperative outcomes in patients with lung cancer.^14^, ^15^ Several previous studies have suggested a complex association between low skeletal muscle mass and physical function.^5^, ^6^, ^16^, ^17^, ^18^

Skeletal muscle mass varies markedly among different age groups, disease types, body sizes, and race/ethnic groups.^19^, ^20^, ^21^, ^22^, ^23^, ^24^, ^25^, ^26^ Given the lack of consensus established cutoff values, the previously used cutoffs for low skeletal muscle mass varied significantly, depending on the specific outcome and the population being studied.^27^ Moreover, the impact of using different thresholds to define low skeletal muscle mass and its relationship with clinical outcomes, such as physical functional limitations (PFLs), remains unknown. Therefore, in this study, focusing on patients with lung cancer, we aimed to evaluate the clinical utility and define diagnostic test metrics for predicting PFLs, using a fixed cutoff value for a CT‐defined low skeletal muscle index (SMI) value from five previous reports. Additionally, we sought to create a simple prediction score incorporating SMI and other clinical factors to predict functional limitations.

## METHODS

### Patients

This cross‐sectional study included 237 patients with lung cancer and was conducted at the Songklanagarind Hospital, a 900‐bed tertiary care university hospital in Hat Yai, Thailand, from August 2021 to December 2023. Patients meeting the inclusion criteria were 18 years or older and had histologically confirmed any‐stage lung cancer diagnoses. All patients were voluntarily enrolled by their chest physicians. Three groups of patients at different stages of lung cancer treatment were enrolled: one group had not yet initiated treatment; the second group was undergoing active treatment and the third group had completed various therapies, such as surgery, concurrent chemoradiotherapy, chemotherapy, or targeted therapy. Patients with large soft tissue metastases at the L3 vertebral level, physical deformities, or neurological deficits and those who refused to undergo the SPPB test were excluded from the study. Baseline data, including the types of treatment and drugs used (for those in the first and second groups), were recorded using the Hospital Information System.

This study was approved by the Human Research Ethics Committee of the Prince of Songhkla University (approval no. REC. 65‐080‐7‐1). Written informed consent was obtained from all the participants.

### Measurement of skeletal muscle mass

**FIGURE 1:**
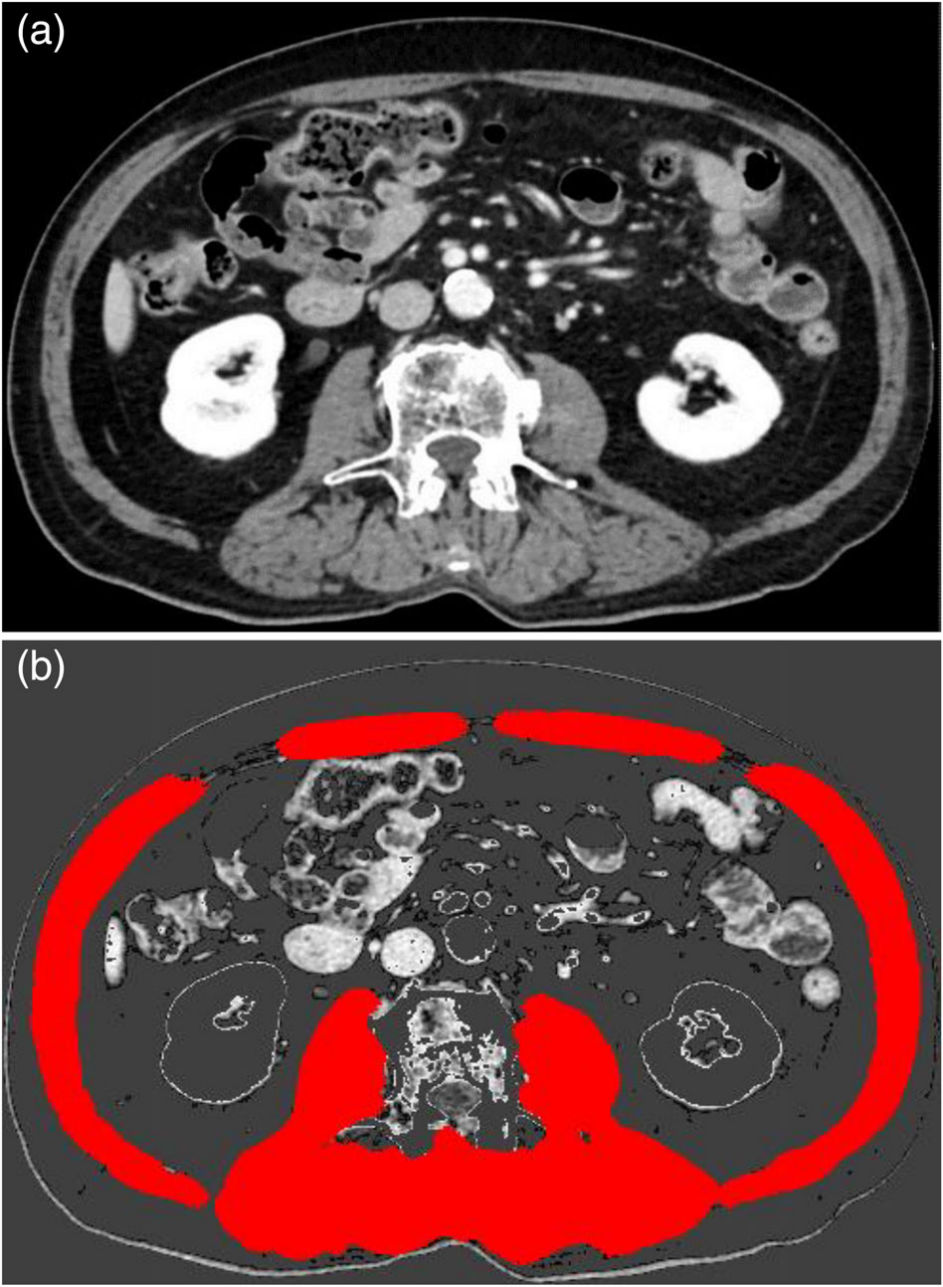
Method for measuring skeletal muscle mass at the third lumbar vertebra. (a) The computed tomography image was taken from the picture archiving and communication system and was saved in digital imaging and communications in medicine (DICOM) format. (b) The image was post‐processed using in‐house developed software for calculation of cross‐sectional area of abdominal wall and back muscles, as delineated in red, to calculate the third lumbar vertebra skeletal muscle area (SMA). The identification and quantification of skeletal muscle relied on Hounsfield unit (HU) thresholds, spanning from −29 to +150 HU.

The SMA at third lumbar vertebral level, determined using CT scans, was used to estimate total body skeletal muscle mass.^8^, ^10^ The SMA was adjusted for height‐squared to calculate the SMI.^21^, ^28^ Single CT slices at the L3 level were carefully chosen and saved as digital imaging and communications in medicine (DICOM) data. Skeletal muscle segmentation was performed using the DICOM data and in‐house software developed using MATLAB (MathWorks) and freeware Python 3.6.13 (Anaconda; https://www.anaconda.com/download). The identification and quantification of skeletal muscle relied on Hounsfield unit (HU) thresholds spanning from −29 to +150 (Figure 1). An experienced technologist manually corrected the outline. The software generated a measurement model based on a previously described deep learning algorithm.^29^, ^30^ This model is a validated model that has been used previously with an accuracy of 99.17%.^31^, ^32^

### Measurement of SPPB

The SPPB test was conducted according to previous guidelines.^33^ During enrollment, the patients completed three evaluations: balance, gait, and a five‐times chair‐stand. Patients were asked to stand with feet side‐by‐side, semi‐tandem, and tandem in order to measure balance, and were asked to walk 4 m at their comfortable pace to assess gait speed. The chair‐stand test was performed by asking the patients to fold their arms and stand up from a seated position five times. In all components of the test, the time required to perform the task was measured, and points were given accordingly. Points were assigned from 0 to 12, where 0 indicated the worst performance, and 12 indicated the best performance. PFLs were defined as SPPB score of ≤9 points.^33^, ^34^

### Statistical analysis

The baseline characteristics are presented with *n* (%) and mean ± standard deviation as appropriate. The chi‐square, Fisher's exact, or Wilcoxon's rank‐sum test was used to calculate differences in baseline data between the patients with or with PFL groups, as appropriate. Frequencies and percentages were used for categorical variables. The diagnostic test metrics of five different cutoff values for identifying low skeletal muscle ranging from 40.8 to 55 cm^2^/m^2^ for males and 31 to 39 cm^2^/m^2^ for females were analyzed.^22^, ^23^, ^24^, ^25^, ^26^ A predictive score was developed using a backward‐stepwise logistic regression model, incorporating potential confounding variables identified in prior studies.^5^, ^6^, ^18^, ^35^, ^36^ The final model included only age and SMI. The model performance was evaluated using the discrimination C‐statistic from bootstrap analysis, Gruppo Italiano per la Valutazione degli Interventi in Terapia Intensiva (GiViTI) calibration belt and decision curve analysis. Comparison of diagnostic performance of previous cutoffs with the prediction score was done using receiver operating characteristic (ROC) curve analysis. The data and graphical figures were analyzed using Stata/MP 18.0 (StataCorp). Statistical significance was set at *p* < 0.05.

## RESULTS

### Patient characteristics

Of the 237 patients, 158 (66.7%) had PFLs (71 males and 87 females; Table 1). Among patients with PFLs, the mean age was 68.8 ± 9.5 years, whereas in patients without PFLs, it was 60.5 ± 10 years, indicating a statistically significant age difference between the groups (*p* < 0.001). The mean body mass index (BMI) of patients with and without PFLs was 22.1 ± 4.7 and 22.9 ± 4.0 kg/m^2^ (*p* = 0.208), respectively. The difference in KPS scores between the two groups was statistically significant (*p* < 0.001), with patients without PFLs having a higher mean KPS score of 95.5 ± 9.1 compared to the mean score of 80.8 ± 19.4 in patients with PFLs.

**TABLE 1:** Patient characteristics.

	No PFL	PFL	Total	*p*‐value
	(*N* = 79)	(*N* = 158)	(*N* = 237)	
Age (years)	60.5 ± 10.0	68.8 ± 9.5	66.0 ± 10.4	<0.001
Sex				0.168
Male	43 (54.4%)	71 (44.9%)	114 (48.1%)	
Female	36 (45.6%)	87 (55.1%)	123 (51.9%)	
BMI (kg/m^2^)	22.9 ± 4.0	22.1 ± 4.7	22.3 ± 4.5	0.208
KPS	95.5 ± 9.1	80.8 ± 19.4	85.7 ± 18.0	<0.001
Treatment status				0.005
Before treatment	40 (50.6%)	107 (67.7%)	147 (62.0%)	
During treatment	6 (7.6%)	17 (10.8%)	23 (9.7%)	
Follow‐up	33 (41.8%)	34 (21.5%)	67 (28.3%)	
Type of treatment				0.611
Chemotherapy	25 (54.3%)	75 (60.5%)	100 (58.8%)	
Targeted therapy	21 (45.7%)	48 (38.7%)	69 (40.6%)	
Immunotherapy	0 (0.0%)	1 (0.8%)	1 (0.6%)	
Line of treatment				0.283
First‐line	36 (78.3%)	78 (62.9%)	114 (67.1%)	
Second‐line	7 (15.2%)	30 (24.2%)	37 (21.8%)	
Third‐line	3 (6.5%)	9 (7.3%)	12 (7.1%)	
Fourth‐line	0 (0.0%)	6 (4.8%)	6 (3.5%)	
Fifth‐line	0 (0.0%)	1 (0.8%)	1 (0.6%)	
Hemoglobin (g/dL)	12.7 ± 1.8	11.6 ± 1.7	12.0 ± 1.8	<0.001
Serum creatinine (mg/dL)	1.0 ± 0.9	0.9 ± 0.3	0.9 ± 0.6	0.141
Albumin (g/dL)	4.0 ± 0.5	3.9 ± 0.8	3.9 ± 0.7	0.915
Skeletal muscle index (cm^2^/m^2^)	39.5 ± 8.4	35.7 ± 7.8	36.9 ± 8.2	<0.001
Skeletal muscle area (cm^2^)	102.7 ± 28.6	89.2 ± 23.8	93.7 ± 26.2	<0.001
Skeletal muscle density (HU)	54.0 ± 7.1	48.8 ± 7.2	50.6 ± 7.6	<0.001

Abbreviations: BMI, body mass index; HU, Hounsfield unit; KPS, Karnosfky performance score; PFL, physical functional limitation.

The distribution of patients across different treatment statuses revealed a significant association between treatment timing and the presence of PFLs (*p* = 0.005). Out of all patients, 147 (62.0%) were enrolled before treatment initiation, with 107 (67.7%) having PFLs and 40 (50.6%) not having PFLs. Among the 23 (9.7%) patients enrolled during treatment, 17 (10.8%) exhibited PFLs, and six (7.6%) did not have PFLs. Additionally, 67 (28.3%) patients were enrolled during follow‐up after treatment, among which 34 (21.5%) had PFLs and 33 (41.8%) did not have PFLs. Chemotherapy was the most frequently used therapy (58.8%), followed by targeted therapy (40.6%), while a small proportion of patients received immunotherapy (0.6%). Further details of drugs and drug combinations used are shown in Table S1.

### CT‐determined skeletal muscle mass

A statistically significant difference was observed in skeletal muscle characteristics between individuals with and without PFLs. Patients with PFLs exhibited lower mean SMI (35.7 ± 7.8 vs. 39.5 cm^2^/m^2^ ± 8.4, *p* < 0.001), SMA (89.2 cm^2^ ± 23.8 vs. 102.7 cm^2^ ± 28.6, *p* < 0.001), and skeletal muscle density (48.8 HU ± 7.2 vs. 54.0 HU ± 7.1, *p* < 0.001) compared to patients without PFLs.

### Diagnostic test metrics

Diagnostic test metrics for predicting PFLs among participants categorized by different SMI cutoff values were performed. When five different cutoffs were used to categorize patients with normal/low SMI, the number of patients with normal SMI, and PFL ranged from 13 to 58, while the number of patients with low SMI, and PFL ranged from 100 to 145 (Table 2). The diagnostic metrics showed suboptimal sensitivity (63.29%–91.77%), specificity (11.39%–50.63%), and area under the receiver operating characteristic curve (AUC) values (0.516–0.592) (Table 3 and Figure 3).

**TABLE 2:** Distribution of number of patients with normal/low SMI and with PFL/without PFL using five previous cutoff values.

Cutoff values		No PFL	PFL	Total
Fearon et al. (2011)^22^ M: 55 cm^2^/m^2^, F: 39 cm^2^/m^2^	Normal SMI	9 (11.4%)	13 (8.2%)	22 (9.3%)
	Low SMI	70 (88.6%)	145 (91.8%)	215 (90.7%)
Kim et al. (2015)^23^ M: 49 cm^2^/m^2^, F: 31 cm^2^/m^2^	Normal SMI	36 (45.6%)	58 (36.7%)	94 (39.7%)
	Low SMI	43 (54.4%)	100 (63.3%)	143 (60.3%)
Kimura et al. (2015)^24^ M: 41 cm^2^/m^2^, F: 38 cm^2^/m^2^	Normal SMI	32 (40.5%)	37 (23.4%)	105 (44.3%)
	Low SMI	47 (59.5%)	121 (76.6%)	132 (55.7%)
Zhuang et al. (2016)^26^ M: 40.8 cm^2^/m^2^, F: 34.9 cm^2^/m^2^	Normal SMI	40 (50.6%)	52 (32.9%)	92 (38.8%)
	Low SMI	39 (49.4%)	106 (67.1%)	145 (61.2%)
Nishikawa et al. (2016)^25^ M: 42 cm^2^/m^2^, F: 38 cm^2^/m^2^	Normal SMI	32 (40.5%)	35 (22.2%)	67 (28.3%)
	Low SMI	47 (59.5%)	123 (77.8%)	170 (71.7%)

Abbreviations: F, female; M, male; PFL, physical functional limitation; SMI, skeletal muscle index.

**TABLE 3:** Diagnostic test metrics for five previous cutoff values.

Diagnostic metrics	Fearon et al. (2011)^22^	Kim et al. (2015)^23^	Kimura et al. (2015)^24^	Zhuang et al. (2016)^26^	Nishikawa et al. (2016)^25^
Sensitivity	91.77%	63.29%	72.02%	67.09%	77.85%
Specificity	11.39%	45.57%	46.34%	50.63%	40.51%
Positive‐predictive value	67.47%	69.93%	76.58%	73.10%	72.35%
Negative‐predictive value	40.87%	38.29%	40.45%	43.48%	47.76%
False‐positive rate	88.60%	54.43%	53.66%	49.37%	59.49%
False‐negative rate	8.22%	36.77%	27.98%	32.91%	22.15%
Overall accuracy	65.01%	57.38%	63.84%	61.52%	65.32%

### Association between SMI and PFL

**FIGURE 2:**
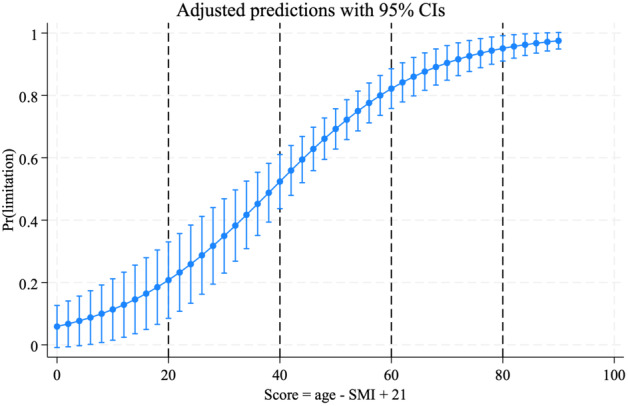
Graph showing the probability of predicting physical functional limitation (y‐axis) using the score (x‐axis). The vertical dotted lines represent the score ranges with its probabilities: < 20 (0.25, 95% CI: 0.03–0.65), 20–40 (0.38, 95% CI: 0.25–0.54), 40–60 (0.72, 95% CI: 0.64–0.80), 60–80 (0.88, 95% CI: 0.73–0.96), and >80 (1.00, 95% CI: 0.29–1.00). CI, confidence interval; Pr(limitation), probability of functional limitation.

**FIGURE 3:**
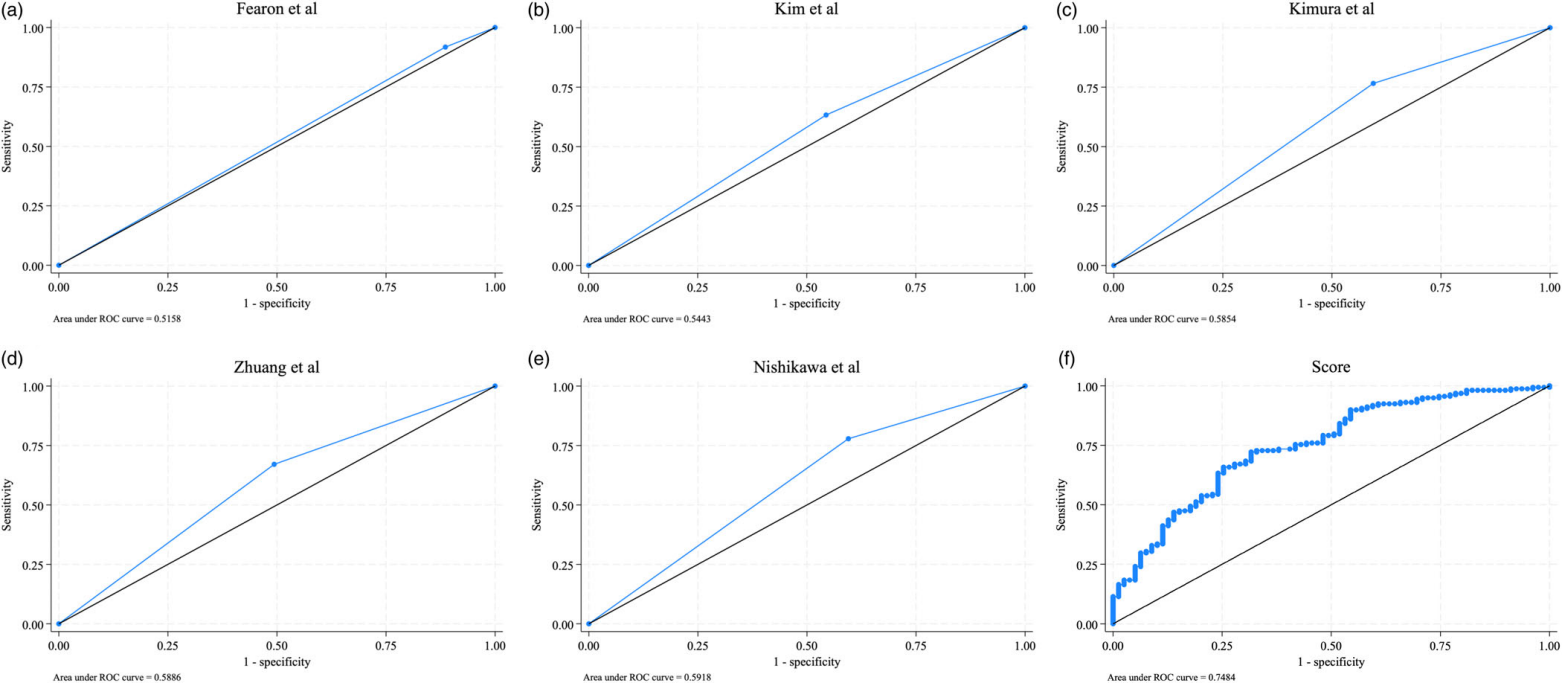
Comparison of ROC curve between previous cutoff values and the prediction score in predicting physical functional limitations. (a) ROC curve for cutoffs proposed by Fearon et al.^22^ (AUC value 0.5158). (b) ROC curve for cutoffs proposed by Kim et al.^23^ (AUC value 0.5443). (c) ROC curve for cutoffs proposed by Kimura et al. ^24^ (AUC value 0.5854). (d) ROC curve for cutoffs proposed by Zhuang et al.^26^ (AUC value 0.5886). (e) ROC curve for cutoffs proposed by Nishikawa et al.^25^ (AUC value 0.5918). (f) ROC curve for the prediction score (AUC value 0.7484). AUC, area under the receiver operating characteristic curve; ROC, receiver operating characteristic curve.

A predictive score was developed using a backward‐stepwise logistic regression model, incorporating age, SMI, sex and BMI, as these variables were found to be associated with physical function in cancer patients.^5^, ^6^, ^18^, ^35^, ^36^ The results indicated that only age (regression coefficient: 0.089, 95% confidence interval [CI]: 0.054–0.123, *p* < 0.001) and SMI (regression coefficient: −0.047, 95% CI: −0.901 to −0.004, *p* = 0.032) were significant predictors of PFLs. Therefore, the final model included only age and SMI, which was used to construct the prediction score (age – SMI + 21). The constant term +21 was added to ensure a more interpretable scoring system that produced scores ranging from 0 to the maximum value. This simplified prediction score was a significant predictor of PFL (odds ratio [OR] = 1.07, 95% CI: 1.05–1.1, *p* < 0.001). The score ranges with corresponding probabilities were < 20 (0.25, 95% CI: 0.03–0.65), 20–40 (0.38, 95% CI: 0.25–0.54), 40–60 (0.72, 95% CI: 0.64–0.80), 60–80 (0.88, 95% CI: 0.73–0.96), and >80 (1.00, 95% CI: 0.29–1.00) (Figure 2). When the prediction score was compared with other cutoff values using ROC curve analysis, it demonstrated a superior AUC value of 0.746 (Figure 3). The discrimination C‐ statistic was 0.747. The GiViTI calibration belt and decision curve analyses agreed well with the prediction score, indicating its accuracy and clinical utility (Figure S1).

## DISCUSSION

The study showed suboptimal diagnostic performance of previously established cutoff values for CT‐determined low skeletal muscle mass in predicting PFLs, with sensitivity varying from 63.29% to 91.77% and specificity ranging from 11.39% to 50.63%. The corresponding AUC values ranged from 0.516 to 0.592, indicating moderate discriminatory ability across the different cutoff values. These findings present insights into the effects of categorizing skeletal muscle mass as “low” or “normal,” which may lead to suboptimal clinical utility, particularly in predicting PFLs. We revealed age and skeletal muscle mass as significant predictors of PFLs (regression coefficient for age: 0.089, *p* < 0.001, regression coefficient for SMI: −0.047, *p* = 0.032). Consequently, we developed a simple prediction score that incorporated the SMI as a continuous variable along with age, which resulted in improved prediction of the outcomes (AUC curve values for prediction score vs. other cutoffs: 0.745 vs. 0.516–0.592).

Most of the previously used thresholds for defining low skeletal muscle mass are based on the outcome parameters and the population being sampled; therefore, the same cutoff values may not apply across different populations or when evaluating different outcomes.^27^ For example, the cutoff points utilized by Kimura et al. was derived from Japanese lung cancer patients with characteristics similar to those in our study, with a focus on its correlation with survival outcomes.^24^ However, when these cutoff values were applied to predict PFLs in the Thai population, the sex‐specific thresholds did not effectively predict the outcome (sensitivity, 72.02%; specificity, 46.34%; AUC, 0.585). This discrepancy supports the concept that variations in the outcome of interest may affect the predictive utility of these cutoff values. In line with our observations, previous reports have indicated that variations in the cutoff values could impact the assessment and comparison of cancer‐related outcomes, such as postoperative complications or overall survival.^37^, ^38^ For instance, as highlighted in a recent meta‐analysis that evaluated postoperative outcomes in lung cancer patients, a study using a higher threshold for the diagnosis of low skeletal muscle mass (male: < 55 cm^2^/m^2^, female: < 39 cm^2^/m^2^) showed no significant difference in the 3‐year overall survival (OS) between patients with and without low skeletal muscle mass (OS rate 83.9% vs. 87.7%, *p* =  0.563).^39^ In contrast, another study using a lower threshold (male: < 43.75 cm^2^/m^2^, female: < 41.10 cm^2^/m^2^) found a significant association with 5‐year OS in the low skeletal muscle mass group (OS rate 85.8% vs. 72.8%, *p* =  0.028).^40^ Beyond these outcomes, low skeletal muscle mass in patients with lung cancer raises concerns regarding chemotherapy toxicity and impaired quality of life.^7^, ^41^ Whether using fixed cutoff values for skeletal muscle mass could also affect the prediction of these outcomes is unclear.

Skeletal muscle mass is a crucial criterion for diagnosing sarcopenia; however, consensus definitions from the Asian Working Groups for Sarcopenia (AGWS) and the European Working Group on Sarcopenia in Older People (EWGSOP) have not specified a cutoff value for CT‐determined skeletal muscle mass. The challenges in determining a single cutoff value arise from the diversity of skeletal muscle mass across different diseases, ethnicities, and sexes.^19^, ^20^, ^21^, ^23^, ^24^, ^25^, ^26^ This results in varying prevalence rates of low skeletal muscle mass; for example, in lung cancer patients, it ranges from 22.4% to 79.2%.^2^, ^23^, ^24^, ^39^, ^42^, ^43^, ^44^, ^45^ Similar findings were observed in this study, in which the prevalence of low skeletal muscle mass ranged from 55.7% to 90.7% when five different cutoff values were used. Importantly, we observed that if we categorized patients based solely on the presence or absence of low skeletal muscle mass, we risked overlooking functional limitations in those without low skeletal muscle mass. For instance, by applying the criteria utilized by Kim et al.,^23^ only 100 of 237 patients were identified as having low skeletal muscle mass and functional limitations. The remaining 58 patients were classified as having normal skeletal muscle mass; however, they exhibited functional limitations (Table 2).

Studies on cancer patients have shown varying relationship between skeletal muscle decline and reduced physical function.^5^, ^6^, ^16^, ^17^, ^18^ A study involving 734 patients with lung cancer found a significant nonlinear association between skeletal muscle mass and physical function.^6^ Similarly, a meta‐analysis of 14 oncological studies (including four studies on lung cancer) found that individuals with low skeletal muscle mass had lower baseline scores in the physical function domains of quality‐of‐life assessments than did those with normal skeletal muscle mass.^18^ Our findings are consistent with these observations. A notable difference in our study was the use of performance‐based measures to assess physical function, with a specific emphasis on higher‐level changes, such as balance and gait speed decline, which can potentially predict treatment outcomes.^14^, ^15^, ^46^ Other studies have predominantly relied on self‐reported measures that are better suited for detecting lower‐level changes, such as the requirement for assistance with daily activities. In contrast to our study, a previous study involving participants with various cancer types found no significant association between skeletal muscle mass, as measured by CT, and overall physical function.^16^ That study utilized both performance‐based assessments, measured using the Timed Up‐and‐Go test and patient‐reported questionnaires to assess physical function. They found a stronger correlation with skeletal muscle density than with muscle mass. These discrepant findings suggest a complex relationship between skeletal muscle mass and physical function, which is not just influenced by muscle mass but also by other factors, such as muscle quality and the age of the patient.

Age, along with skeletal muscle mass, emerged as a significant predictor of declining physical function. This aligns with a previous study in lung cancer patients aged over 70 years that investigated the association between physical function, measured by handgrip strength, and the incremental shuttle walking distance, and demonstrated a linear relationship between skeletal muscle decline and reduced physical function.^5^ An age‐related decline in skeletal muscle mass and physical function in the general population is well‐documented.^47^, ^48^, ^49^ Furthermore, existing reports indicate an expedited decline in skeletal muscle mass and physical function among older patients with cancer compared with the general aging population.^11^ This emphasizes the challenges faced by older patients with lung cancer, who experience the combined effects of aging and cancer on the development of sarcopenia. Therefore, in the context of lung cancer patients, we propose the use of our simple prediction score (age ‐ SMI + 21), which offers a more comprehensive means of identifying physical functional limitations using skeletal muscle mass.

This study had some limitations. To maintain statistical power, certain important predictors of low skeletal muscle mass and physical function, such as comorbidities, disease stage, type of cancer treatment, treatment status and skeletal muscle density, were not included in our final analysis. Additionally, our study included patients undergoing various treatments at different disease stages, precluding the evaluation of different treatment effects, such as chemotherapy or targeted therapy. As a cross‐sectional study, our research could not establish causation, and does not yield insight into temporal changes in skeletal muscle mass and physical function. A notable strength of our study was the use of CT to measure skeletal muscle mass, which is a standard method in oncology patients, ensuring precise measurements. Moreover, the physical function assessment employed in our study was a reliable and widely used performance‐based evaluation tool that enhanced the validity of our findings.

In conclusion, the use of fixed cutoffs for CT‐defined low skeletal muscle mass diagnosis may not effectively predict PFL, potentially overlooking declining physical functions in patients with lung cancer. Our findings demonstrated that incorporating age and utilizing the SMI as a continuous variable enhances predictive accuracy for PFLs. As the relationship between skeletal muscle mass and PFLs is complex, future studies should explore additional factors, such as the impact of different treatment types, treatment status, skeletal muscle density and stage of lung cancer.

## AUTHOR CONTRIBUTIONS

All authors had unrestricted access to the study data and were responsible for data integrity and accuracy. Study design: Wiwatana Tanomkiat, Shiva Raj Timsina and Wiwatana Tanomkiat and Sarayut L. Geater. Methodology: Shiva Raj Timsina, Sarayut L. Geater, Natee Ina and Wiwatana Tanomkiat. Data collection and recording: Shiva Raj Timsina, Sarayut L. Geater and Natee Ina. Data analysis: Shiva Raj Timsina and Sarayut L. Geater. Resources: Wiwatana Tanomkiat, Sarayut L. Geater and Natee Ina. Writing ‐ original draft: Shiva Raj Timsina. Writing ‐ review and editing: Shiva Raj Timsina, Wiwatana Tanomkiat, Sarayut L. Geater and Natee Ina. Supervision: Wiwatana Tanomkiat, Sarayut L. Geater and Natee Ina.

## CONFLICT OF INTEREST STATEMENT

None of the authors have any conflict of interest to declare.

## Supporting information


**Figure S1.** (a) GiViTI calibration belt and (b) decision curve analysis demonstrates good agreement with the prediction score, indicating its accuracy and clinical utility.

**Table S1.** Name of drugs or drug combinations used (for those in the first and second groups).

## Data Availability

The datasets used in this study are available from the corresponding author upon request.
