# Supplementary material for: Exploring previously used thresholds for computed tomography‐defined low skeletal muscle mass in predicting functional limitations among lung cancer patients
Source: Thorac Cancer. 2024 Apr 26;15(16):1287–95. doi: 10.1111/1759-7714.15313 (PMC11147667; doi:10.1111/1759-7714.15313)
Supplement: Supplementary file 1 — Figure S1. (a) GiViTI calibration belt and (b) decision curve analysis demonstrates good agreement with the prediction score, indicating its accuracy and clinical utility. [file TCA-15-1287-s001.pdf]

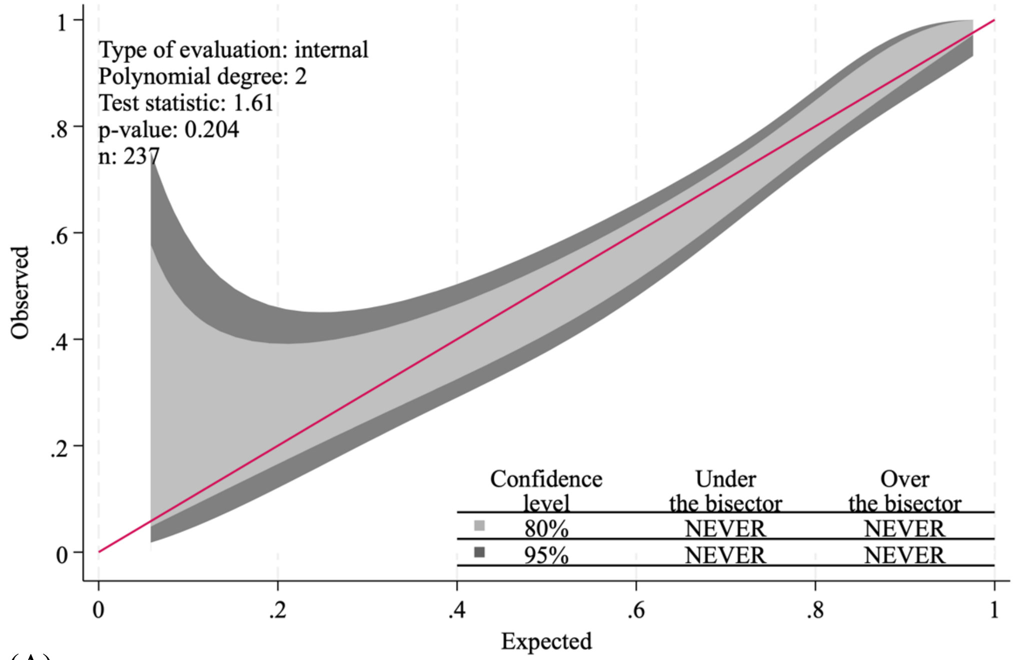

(A)

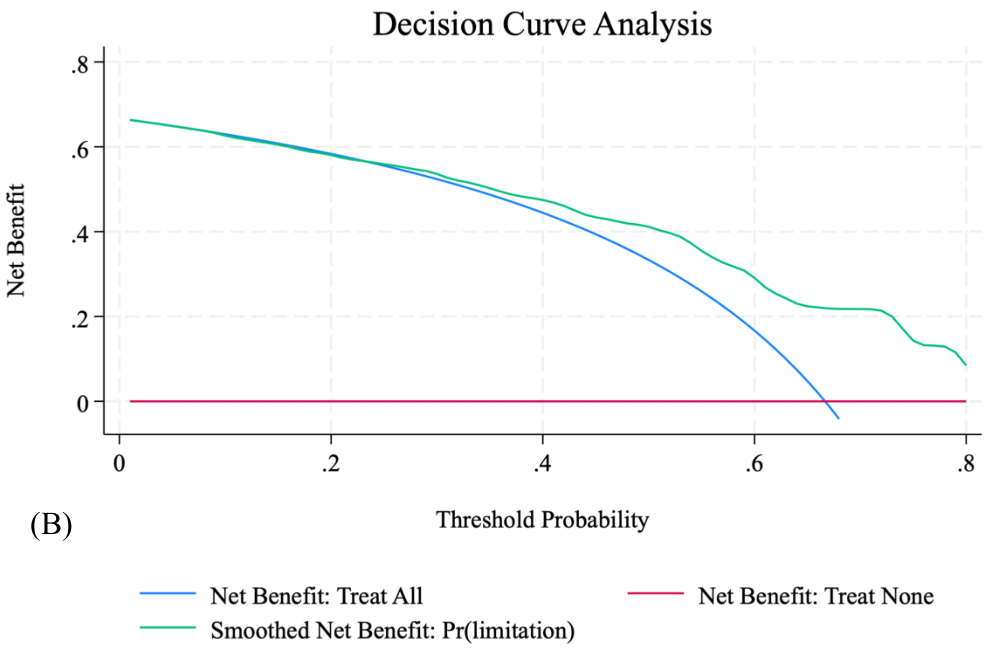

(B)

Figure S1. (A) GiViTI Calibration Belt and (B) decision curve analysis demonstrates good agreement with the prediction score, indicating its accuracy and clinical utility.
