# Supplementary material for: Exploring previously used thresholds for computed tomography‐defined low skeletal muscle mass in predicting functional limitations among lung cancer patients
Source: Thorac Cancer. 2024 Apr 26;15(16):1287–95. doi: 10.1111/1759-7714.15313 (PMC11147667; doi:10.1111/1759-7714.15313)
Supplement: Supplementary file 2 — Table S1. Name of drugs or drug combinations used (for those in the first and second groups). [file TCA-15-1287-s002.docx]

**SUPPORTING INFORMATION**

Table S1. Name of drugs or drug combinations used (for those in the first and second groups).

|  | No PFL | PFL | Total |
| --- | --- | --- | --- |
|  | (N=46) | (N=124) | (N=170) |
| Name of therapy |  |  |  |
| Afatinib | 0 (0.0%) | 1 (0.8%) | 1 (0.6%) |
| Atezolizumab | 0 (0.0%) | 1 (0.8%) | 1 (0.6%) |
| CAV | 1 (2.2%) | 1 (0.8%) | 2 (1.2%) |
| Cb Eto | 0 (0.0%) | 1 (0.8%) | 1 (0.6%) |
| Cb Eto Atezo | 1 (2.2%) | 0 (0.0%) | 1 (0.6%) |
| Ceritinib | 5 (10.9%) | 3 (2.4%) | 8 (4.7%) |
| Cis Eto | 4 (8.7%) | 5 (4%) | 9 (5.3%) |
| Crizotinib | 2 (4.3%) | 3 (2.4%) | 5 (2.9%) |
| Docetaxel | 4 (8.7%) | 17 (13.7%) | 21 (12.4%) |
| Erlotinib | 10 (21.7%) | 29 (23.4%) | 39 (22.9%) |
| Gefitinib | 1 (2.2%) | 2 (1.6%) | 3 (1.8%) |
| Gem Cis | 0 (0.0%) | 3 (2.4%) | 3 (1.8%) |
| Osimertinib | 3 (6.5%) | 10 (8.1%) | 13 (7.6%) |
| Pac Cb | 15 (32.6%) | 43 (34.7%) | 58 (34.1%) |
| Peme Cb Pemb | 0 (0.0%) | 1 (0.8%) | 1 (0.6%) |
| Pemetrexed | 0 (0.0%) | 4 (3.2%) | 4 (2.4%) |

PFL= Physical functional limitation; CAV = cyclophosphamide, doxorubicin, vincristine; Cb Eto = carboplatin, etoposide; Cb Eto Atezo = carboplatin, etoposide, atezolizumab; Cis Eto = cisplatin, etoposide; Gem Cis = gemcitabine, cisplatin; Pac Cb = paclitaxel, carboplatin; Peme Cb Pemb = pemetrexed, carboplatin, pembrolizumab.
